# Foot and ankle characteristics associated with fear of falling and mobility in community-dwelling older people: a cross-sectional study

**DOI:** 10.1186/s13047-022-00593-w

**Published:** 2022-12-09

**Authors:** Fateme Pol, Zahra Khajooei, Sayed Mohsen Hosseini, Alireza Taheri, Saeed Forghany, Hylton B. Menz

**Affiliations:** 1 Musculoskeletal Research Center, Isfahan University of Medical Sciences, Isfahan, Iran; 2 Epidemiology and Biostatistics Department, Isfahan University of Medical Sciences, Isfahan, Iran; 3 Health Sciences Research Centre, University of Salford, Greater, Manchester, UK; 4 Discipline of Podiatry, School of Allied Health, Human Services and Sport, La Trobe University, Melbourne, VIC 3086 Australia

**Keywords:** Fear of falling, Foot, Mobility, Older people

## Abstract

**Background:**

Fear of falling is multifactorial in etiology and is associated with falls. It has been demonstrated that foot problems increase the risk of falls in older people. Therefore, the objective of this study was to investigate the associations of foot and ankle characteristics with fear of falling and mobility in community-dwelling older people.

**Method:**

One hundred and eighty-seven community-dwelling older adults (106 females) aged 62–90 years (mean 70.5 ± 5.2) from Isfahan, Iran, were recruited. Foot and ankle characteristics (including foot posture, range of motion, muscle strength, deformity, tactile sensation, pain and dynamic function), fear of falling (Fall Efficacy Scale International) and mobility (Timed Up and Go Test) were measured. Two multivariate linear regression analyses identified variables independently associated with fear of falling and mobility.

**Results:**

Linear regression analysis revealed that less ankle plantarflexor muscle strength, greater pressure-time integral, foot pain, and reduced tactile sensitivity of the ankle were significantly and independently associated with increased fear of falling. The total variance explained by the model was 59%. Less ankle plantarflexor muscle strength, greater pressure-time integral, and slower centre of pressure velocity were significantly and independently associated with poorer mobility. The total variance explained by the model was 48%.

**Conclusion:**

Several foot and ankle characteristics are associated with fear of falling and mobility in older people. Targeting these modifiable risk factors may play a role in reducing fear of falling and enhancing mobility performance in this population.

## Background

Fear of falling has been described as ongoing concern about falling that ultimately limits the performance of daily activities [1]. Fear of falling is multifactorial in etiology, and common among community-dwelling older adults, with prevalence ranging from 26 to 55% [2]. Fear of falling is associated with falls, although the causal relationship is unclear and may be bi-directional, as the two outcomes share risk factors [3]. One such risk factor is foot structure and function. The foot provides the only contact source while standing and walking, and it has been demonstrated that foot problems increase the risk of falls in older people [4, 5]. However, the associations between foot characteristics, fear of falling and mobility have not been examined in detail.

Three studies have explored the association between foot characteristics and fear of falling. Binda et al [6] compared 40 older people with and without fear of falling and found that the fearful group had significantly weaker ankle joint plantarflexors. Harada et al [7] conducted a cross-sectional study in a large (*n* = 10,581) sample of community-dwelling elderly people and reported significant associations between fear of falling and skin and nail problems affecting the foot. More recently, Muchna et al [8] reported that fear of falling was associated with foot problems (defined as self-reported foot pain, peripheral neuropathy, or deformity) in 128 community-dwelling older people. Although these studies provide some evidence of an association between foot characteristics and fear of falling, they have used different tools to document fear of falling, and have used only a limited selection of foot and ankle measures.

Therefore, building on our previous work [5], this study was conducted to determine whether a comprehensive range of foot characteristics (covering the domains of foot posture, muscle strength, range of motion, tactile sensitivity, deformity, foot pain, and plantar pressure) have an association with fear of falling and mobility impairment in community-dwelling older people.

## Methods

### Ethical approval

Ethical approval was granted by the Isfahan University of Medical Sciences (IR.MUI.RESEARCH.REC.1398.451) and informed consent was obtained from all persons before their participation.

### Recruitment

Participants were recruited from 15 health centers (one representative of each urban area) of Isfahan, Iran between April and November, 2020. Older people were deemed ineligible for the study if they were unable to ambulate for at least 10 m without an assistive device, scored *<* 7 for the Short Portable Mental Status Questionnaire, had diabetic foot syndrome, neurological diseases, or previously had lower extremity surgery. From the 984 potential participants, 584 met the inclusion criteria according to the information provided in their medical records. Following more detailed telephone screening, 240 were deemed eligible. These individuals were then randomly selected and contacted by telephone and invited into the study. If they declined or were ineligible, the next person from the randomisation list was contacted until the sample size was met.

### Sample size

Sample size (n) was calculated using the sample size formula:

$$\mathrm n=\frac{z_{\mathit1\mathit-\alpha\mathit/\mathit2}^{\mathit2}p\left(\mathit1\mathit-p\right)}{d^{\mathit2}}$$

…where z is the confidence level (1.96 standard deviations; 95%), α is the alpha level (0.05), $p$ is the estimated probability of having a foot problem (0.45) and $d$ is the precision (0.06). This calculation identified that 264 participants would need to be recruited. However, due to the impact of the coronavirus pandemic, recruitment had to cease at 225 participants (94% of the eligible sample), and of these, 38 cases were excluded due to errors in the plantar pressure data. Therefore, the final study sample comprised 187 community dwelling older people (106 females) aged 62–90 years (mean 70.5 ± 5.2, body mass index 27.7 ± 4.1).

### Foot and ankle structural and functional characteristics

Structural and functional characteristics of the foot and ankle were tested across the domains of foot posture, muscle strength, range of motion, tactile sensitivity, deformity, foot pain, and plantar pressure.

Foot posture was assessed using foot posture index (FPI), arch index (AI), and normalised navicular height truncated (NNHt). The FPI involved the rating of 6 criteria, the sum of which provided a single index of the pronated/supinated foot posture. The AI was calculated by the ratio of midfoot area to the foot (excluding toes) using the EMED pressure plate (novelGmbH, Munich, Germany, 2 sensor/cm^2^, 50Hz). NNHt represents the navicular height divided by truncated foot length (i.e. foot length excluding the toes) in bipedal standing.

Isometric muscle strength of the ankle (dorsiflexion, plantar flexion, inversion, and eversion) was measured with a hand-held dynamometer (Digital Force Gauge 5kg; accuracy 0.001 kg) mounted on an apparatus to ensure isometric contraction via the make-test. Hallux and lesser toe muscle strength were quantified using a previously developed protocol. Participants were instructed to push down as hard as possible on an EMED pressure platform. The test protocols have been described in detail elsewhere [5].

Two measures of foot and ankle passive range of motion, passive ankle dorsiflexion and hallux first metatarsophalangeal (MTP) joint extension, were performed via a single standard video camera (50 Hz, 25 fps) which was placed perpendicular to the plane of motion and three skin markers (based on the same goniometry protocol) employed to capture the range of motion. Frame by frame advance was used to identify the instance of the maximum range of motion. Kinovea software (https://www.kinovea.org/) was used to measure these angles. Tactile sensitivity at the ankle was assessed using a single Semmes–Weinstein-type pressure monofilament. The monofilament was applied three times to the lateral malleolus of the ankle while the participant kept their eyes closed.

Hallux valgus was documented using the Manchester scale, a reliable and valid clinical tool based on four photographs of the foot [9]. The presence of foot pain was determined with the Manchester Foot Pain and Disability Index (MFPDI) [10].

Foot function was assessed using barefoot plantar pressure analysis using the EMED-le pressure plate. Three trials were recorded for each participant’s dominant limb with a two-step gait initiation protocol at a comfortable walking speed. Following data collection, the novel scientific software v23 was used to calculate the pressure-time integral in the total foot, centre of pressure (COP) velocity of the total foot, and the centre of pressure excursion index (CPEI), a measure of the mediolateral shift in COP throughout the gait cycle.

### Fear of falling and mobility assessment

The Falls Efficacy Scale International (FESI) was used to assess the level of concern of falling during 16 activities of daily living, including social activities that may contribute to the quality of life [11]. The level of concern for each item was scored using a four-point scale (1 = not at all concerned, 4 = very concerned) within a total score range of 16–64. Mobility was assessed using the Timed Up and Go (TUGT) test by measuring the time taken for the participant to stand up from a chair, walk 3m, turn around, walk back, and sit down. Three trials were recorded for each participant and averaged. The TUGT has good validity and reliability as a measure of mobility [12].

### Statistical analysis

Statistical analyses were carried out using IBM Statistical Package for the Social Sciences (SPSS) (IBM Corporation, Armonk, NY) with a 5% level of significance. Descriptive statistics were used to provide an overview of the demographic variables. The Kolmogorov–Smirnov test was used to check the normality of the data. Pearson’s *r* linear correlation was used where the relationship between normally distributed parameters was assessed. Otherwise, Spearman’s rho correlation test was used. Independent samples t-tests were performed to evaluate differences in FESI and TUGT according to dichotomous variables. All variables significantly associated with the dependent variable were entered in two stepwise multiple linear regression models to determine their relative importance in explaining variance in FESI and TUGT. Variables were selected based on the results of the correlation results (*r >* 0.3) or t-test (*p* < 0.2) for dichotomous variables. Preliminary analyses were conducted to ensure no violation of the assumption of normality, linearity, multicollinearity, and homoscedasticity. The overall fit of linear regression models was quantified by *R*^2^.

## Results

### Participant characteristics

Of the 187 participants recruited into the study, 109 (58.6%) had poor visual acuity (using a 10% low-contrast letter chart), 40 (21.5%) used psychoactive medications, and 95 (51.1%) reported taking > 4 medications per day (Table 1).

**Table 1:** Demographics, medical history, fear of falling and mobility of study population. Data presented as mean (SD) unless specified

Demographics	
Participants, n	187
Sex, n (% female)	106 (56.7)
Age, years	70.52 (5.2)
BMI, kg/m^2^	27.74 (4.1)
Medical history	
Poor vision (low contrast visual acuity), n (%)	109 (58.6)
Four or more medications excluding vitamins, n (%)	95 (51.1)
Medications (any psychotropic), n (%)	40 (21.5)
Fear of falling and mobility	
Fall Efficacy Scale International	27.01 (10.1)
Timed Up and Go Test	11.78 (3.27)

*Abbreviations: BMI* Body Mass Index, *kg/m*^*2*^ kilogram per square metre

### Descriptive statistics

Descriptive statistics for each of the foot and ankle characteristics are shown in Table 2.

**Table 2:** Descriptive statistics for foot and ankle characteristics. Data presented as mean (SD) unless specified

Domain	Variable (unit)	Mean (SD)	Range
Foot posture	FPI	4.20 (3.1)	-3-12
	Arch index	0.23 (0.03)	0.16–0.34
	NNHt	0.26 (0.03)	0.19–0.36
Muscle strength	Plantarflexor (kg)	10.46 (1.5)	7.77–15.85
	Dorsiflexor (kg)	8.09 (1.24)	5.33–12.92
	Invertor (kg)	5.98 (1.09)	3.67–8.94
	Evertor (kg)	5.12 (1.10)	3.35–14.95
	Hallux (%BW)	11.77 (5.98)	6-26.56
	Lesser toe (%BW)	8.66 (3.82)	2.43–22.29
Range of motion	Ankle dorsiflexion (°)	15 (7.04)	2-34.67
	First MTP extension (°)	47.55 (6.22)	29.67-66
Peripheral sensation	Ankle tactile sensation, n (%)	51 (27.3)	-
Deformity	Hallux valgus, n (%)	77 (41.2)	-
Foot pain	MFPDI	7.66 (8.8)	0–34
Plantar pressure	CPEI (%)	16.42 (6.64)	0.73–31.09
	Pressure-time integral (%BW*S/cm^2^)	3.44 (1.244)	1.52–7.92
	COP velocity (cm/s)	19.71 (402)	11–29

*Abbreviations: FPI* Foot posture index, *NNHt* Normalized navicular height truncated, *MTP* Metatarsophalangeal, *MFPDI* Manchester Foot Pain Diasability Index, CPEI Centre of pressure excursion index, *kg* kilogram, BW Bodyweight, BW*S/cm^2^ Body weight second per square centimeter, COP Centre of pressure,  *cm/s* centimeter per second

### Univariate analysis: associations of foot and ankle characteristics with fear of falling and mobility

Tables 3 and 4 show the associations between the foot and ankle characteristics with fear of falling and mobility performance on continuous (Pearson’s *r*) and dichotomous variables (independent sample *t*-tests), respectively. Independent sample *t*-tests revealed that participants who failed the ankle tactile sensation and vision test and use > 4 medications per day scored worse on FESI and TUGT (*p* < 0.001). FESI scores were worse in older adults who used psychoactive medications (*p* = 0.06).

**Table 3:** Associations between fear of falling and mobility with foot and ankle characteristics (Pearson’s r)

Domain	Variable	FESI	TUGT
Foot posture	FPI	-0.05	0.0
	Arch index	-0.11	-0.11
	NNHt	-0.03	0.06
Muscle strength	Plantarflexor	-0.49	-0.45
	Dorsiflexor	-0.45	-0.46
	Invertor	-0.45	-0.39
	Evertor	-0.29	-0.24
	Hallux	-0.13	-0.2
	Lesser toe	-0.16	-0.22
Range of motion	Ankle dorsiflexion	-0.18	-0.26
	First MTP joint extension	-0.35	-0.3
Foot pain	MFPDI	0.58	0.38
Plantar pressure	Pressure-time integral	0.34	-0.3
	COP velocity	-0.36	-0.39
	CPEI	-0.17	-0.18

*Abbreviations: FESI* Falls Efficacy Scale International, *TUG* Timed Up and Go Test, *FPI* Foot posture index, *NNHt* Normalized navicular height truncated, *MTP* Metatarsophalangealt, *MFPDI* Manchester Foot Pain and Disability Index, *COP* Centre of pressure, *CPEI* Centre of pressure excursion index

**Table 4:** Fear of falling and mobility test scores according to dichotomous variables

Domain	Variable		FESI Mean (SD)	*P*-value	TUGT Mean (SD)	*P*-value
Deformity	Hallux valgus	Yes (*n* = 109)	12.23 (3.28)	0.11	25.68 (10.3)	0.03
		No (*n* = 77)	11.46 (3.24)		28.89 (9.57)	
Peripheral sensation	Ankle tactile	Yes (*n* = 51)	33.58 (9.92)	< 0.001	13.27 (2.88)	< 0.001
		No (*n* = 135)	24.53 (9.04)		11.21 (3.24)	
Medications	Psychoactive	Yes (*n* = 40)	29.67 (10.43)	0.06	11.56 (2.92)	0.63
		No (*n* = 146)	26.28 (9.93)		11.84 (3.37)	
Vision	Poor vision	Yes (*n* = 109)	28.67 (10.16)	< 0.001	12.65 (3.4)	< 0.001
		No (*n* = 77)	24.66 (9.60)		10.54 (2.50)	

*Abbreviations: FESI* Falls Efficacy Scale International, *TUG* Timed Up and Go Test

### Multivariate analysis: associations of foot and ankle characteristics with fear of falling and mobility

Multivariate linear regression examined the associations of foot and ankle characteristics with fear of falling and mobility in two linear regression models (Table 5). The first model examined foot and ankle structural and functional characteristics (using a stepwise method) with fear of falling. Eleven continuous (*r* > 0.3) and dichotomous variables (*p* < 0.2) were considered in the first regression model, including plantar flexor and invertor muscle strength, first MTP joint ROM, PTI, COP velocity, MFPDI, ankle tactile sensitivity, hallux valgus, vision, use of psychoactive medications and using four or more medications. To avoid multicollinearity, dorsiflexor muscle strength was excluded from the model as it was highly correlated with plantarflexor muscle strength (*r* = 0.7). Plantarflexor muscle strength, PTI, ankle tactile sensitivity in addition to foot pain were independently associated with fear of falling. The total variance explained by the model was 59% (F _5, 158_= 46.19, *p* < 0.001).

**Table 5:** Linear regression analysis showing an association between fear of falling and mobility (dependent variables) and foot and ankle characteristics (independent variables)

	Fear of falling (FESI)			
R^2^	0.59			
Independent variable	β	SE	Beta	*p*-value
PTI	1.08	0.43	0.13	0.01
Plantarflexor muscle strength	-1.31	0.36	-0.2	< 0.001
MFPDI	0.45	0.06	0.39	< 0.001
Tactile sensitivity test	4.60	1.20	0.2	< 0.001
Medications (four or more excluding vitamins)	4.9	1.15	0.24	< 0.001
	Mobility (TUGT)			
R^2^	0.48			
PTI	0.38	0.19	0.14	0.04
Plantarflexor muscle strength	-0.35	0.14	-0.17	0.01
COP velocity total foot	-0.002	0.001	-0.26	< 0.001
Vision	1.15	0.38	0.17	0.003
Medications (four or more excluding vitamins)	0.88	0.44	0.13	0.04
FESI	0.11	0.02	0.36	< 0.001

*Abbreviations: β* unstandardized coefficient, *Beta* standardized coefficient, MFPDI Manchester Foot Pain and Disability Index, *PTI* Pressure-time integral, *COP* Centre of pressure, *FESI* Falls Efficacy Scale International

For the second linear regression model, the same procedure was performed with the TUGT score as the dependent variable. In addition to the previous independent variables, the FESI score was entered in the model, and the use of psychoactive medications was excluded. Plantarflexor muscle strength, PTI, and COP velocity were independently associated with the TUGT score. Total variance explained by the model was 48% (F _6, 157_= 24.86, *p* < 0.001).

## Discussion

The present study examined the association between foot and ankle characteristics with fear of falling (assessed using the Falls Efficacy Scale International [FESI]) and mobility (assessed using the Timed Up and Go Test [TUGT]) in older adults. The findings of this study indicate that foot and ankle characteristics contribute to both fear of falling and impaired mobility in older adults. Our results revealed that decreasing plantar flexor muscle strength and increasing pressure-time integral, foot pain, and tactile sensitivity were significantly and independently associated with increased fear of falling. We also observed independent associations between decreased plantar flexor muscle strength and increased pressure-time integral and decreased centre of pressure velocity with impaired mobility.

Overall functioning of the postural control system is dependant on lower limb muscle strength [13]. The foot and ankle complex, as the only part of the body that contacts the ground during walking and standing, needs to be sufficiently stable to maintain the center of mass within the base of support. Foot and ankle muscles contribute to postural stability [14], so decreased strength and the subsequent perceived loss of balance in daily life situations may induce fear of falling. The results of our study confirm that leg muscles play an important role in balance as ankle plantarflexor muscle strength was independently and significantly associated with FESI and TUGT. This finding is consistent with the available literature showing that those with fear of falling demonstrate less strength of the knee extensor [6, 15, 16] and ankle plantarflexor [6] muscles.

The association between plantar loading patterns during walking with fear of falling and mobility performance is a novel finding. The significant association between increased pressure-time integral with higher FESI and TUGT scores is particularly noteworthy. The underlying mechanism for this is difficult to determine, but the greater duration and magnitude of plantar loading when walking may predispose to foot pain, which has been shown to impair balance and functional ability in older people [17–19]. Alternatively, an increased PTI may be a reactive response to perceived instability to increase the duration of foot contact with the ground.

Although pain has previously been reported to be associated with impaired balance and fear of falling, most studies have investigated general body pain [20–22]. We found foot pain to be independently associated with fear of falling, which is consistent with the only other study in which this association was investigated [8]. Clinicians working with older adults with pain should therefore consider foot pain as an individual risk factor for fear of falling.

Older adults who failed the tactile sensitivity test scored worse in FESI, and the results of the linear regression model showed that it has a strong association with the FESI score. The integration of visual, vestibular, and somatosensory information is necessary to generate appropriate balance responses [23]. Individuals rely primarily on proprioceptive and cutaneous input to maintain standing balance [24], and several studies have shown that age-related peripheral sensory loss is associated with increased postural sway [25, 26] and is an independent predictor of falls [27, 28]. The perceived loss of balance due to impaired tactile sensitivity may therefore induce fear of falling in older people.

The centre of pressure (COP) on the plantar surface of the foot reflects the progression of the whole body center of mass during gait [29], therefore the forward velocity of the COP may potentially affect walking speed and sit to stand, the two components of TUGT. The negative association we observed between COP velocity and higher TUGT scores (indicative of poorer mobility) suggest that both walking speed and sit to stand, to some extent, require the same strategies in which the goal is to regulate speed and to remain upright during the transfer of bodyweight. This observation is consistent with previous studies which have reported that both walking [30] and sit to stand [31] are skills that involve rapid and coordinated balance transfers.

A number of potential limitations need to be considered. Firstly, the cross-sectional design of this study does not provide evidence of temporal relationships between variables, although it is unlikely that these foot characteristics are the result of either fear of falling or TUGT. In addition, the recruitment strategy involved inviting older people to participate in a study of foot and ankle assessment and association with fear of falling, so the prevalence of foot problems may be overestimated compared to the general older population. Secondly, we used the TUGT as our measure of mobility. Although this is a widely used and useful measure, other tests more specific to balance may have identified stronger associations with foot and ankle characteristics. Finally, although we used a wide range of foot and ankle measures, inclusion of other explanatory variables (such as anxiety and depression scales and measures of frailty) may have explained additional variance and enhanced the model [32].

## Conclusion

Foot and ankle characteristics, in particular plantarflexor weakness and higher plantar pressures when walking, are associated with fear of falling and mobility in community-dwelling older people. Given that these characteristics are modifiable, clinical interventions such as foot strengthening programs, footwear and foot orthoses may play a role in reducing fear of falling and optimising mobility in this age-group when combined with other targeted interventions such as medication review, home hazard modification and balance exercises.

## Data Availability

The datasets used and/or analysed for this study are available from the corresponding author upon reasonable request.
